# The graft versus leukemia effect: donor lymphocyte infusions and cellular therapy

**DOI:** 10.3389/fimmu.2024.1328858

**Published:** 2024-03-15

**Authors:** Katie Maurer, Joseph H. Antin

**Affiliations:** Division of Hematologic Malignancies, Department of Medical Oncology, Dana-Farber Cancer Institute, Harvard Medical School, Boston, MA, United States

**Keywords:** GVL, graft versus leukemia, DLI, donor lymphocyte infusion, cellular therapy

## Abstract

Allogeneic hematopoietic stem cell transplantation (HSCT) is a potentially curative therapy for many hematologic malignancies as well as non-malignant conditions. Part of the curative basis underlying HSCT for hematologic malignancies relies upon induction of the graft versus leukemia (GVL) effect in which donor immune cells recognize and eliminate residual malignant cells within the recipient, thereby maintaining remission. GVL is a clinically evident phenomenon; however, specific cell types responsible for inducing this effect and molecular mechanisms involved remain largely undefined. One of the best examples of GVL is observed after donor lymphocyte infusions (DLI), an established therapy for relapsed disease or incipient/anticipated relapse. DLI involves infusion of peripheral blood lymphocytes from the original HSCT donor into the recipient. Sustained remission can be observed in 20-80% of patients treated with DLI depending upon the underlying disease and the intrinsic burden of targeted cells. In this review, we will discuss current knowledge about mechanisms of GVL after DLI, experimental strategies for augmenting GVL by manipulation of DLI (e.g. neoantigen vaccination, specific cell type selection/depletion) and research outlook for improving DLI and cellular immunotherapies for hematologic malignancies through better molecular definition of the GVL effect.

## 1 Introduction

The graft versus leukemia (GVL) effect of allogeneic hematopoietic stem cell transplantation (HSCT) is a clinically well-established phenomenon reliant upon the donor immune system recognizing and eliminating cancerous cells. Effective GVL is a key component of the curative potential of HSCT for hematologic malignancies, yet the biological basis for GVL remains poorly understood, thus limiting the ability to leverage this phenomenon for therapeutic benefit. Anti-cancer immune reactions were clearly observed in animal models of leukemia as early as the 1960s (1), though early immunotherapy trials in humans in the 1980s were underwhelming (2). GVL reactions in humans first gained support in 1990 when Horowitz et al. demonstrated that recipients of allogeneic stem cell grafts depleted of T cells had a higher incidence of relapse (3). This study also found that recipients of syngeneic grafts (i.e. stem cells from an identical twin) had a higher relapse rate compared to recipients of allografts (3). Both lines of evidence support the notion that the allogeneic donor immune system helps to provide immunologic control of disease.

In the modern era, GVL activity is clearly observed in two clinical scenarios of incipient relapse (e.g. recurrence of disease-associated mutations, decreasing cell counts, loss of donor chimerism) or overt relapse. These can be addressed by tapering of immune suppression (IST) and/or donor lymphocyte infusions (DLI). For patients with relapsing disease, immunosuppressive medications for prevention of graft versus host disease (GVHD) can be rapidly tapered, thereby engendering enhanced donor immune activation, primarily T cell alloreactivity. In approximately 20% of patients, IST alone is sufficient to reinduce sustained remission (4). DLI on the other hand involves reinfusion of peripheral blood lymphocytes from the original donor into the HSCT recipient. DLI was first shown to be successful for reinducing remission in patients with relapsed hematologic malignancy in 1990 (5) and since that time has become an established mode of adoptive cellular immunotherapy.

In this review, we will discuss the establishment of DLI as the archetype of effective GVL, its role and efficacy in different hematologic malignancies, clinical applications and combination therapies for enhancing the GVL effect of DLI, and recent biological insights into the mechanisms underlying the success of this therapy.

## 2 Section 1 – historical context of GVL and role of DLI

One year before E. Donnall Thomas’s seminal work describing the first successful bone marrow transplantation in humans (6), Barnes and Loutit observed the first evidence of GVL activity in rodents (7). Mice treated with radiation doses insufficient to eradicate leukemia cells had disease recurrence if reconstituted with bone marrow stem cells from the same strain but maintained remission if given stem cells from a different strain, thus implicating the donor immune system in elimination of residual leukemia and maintenance of remission. As bone marrow transplantation gained traction for its potential as a curative therapy, the presence of GVL activity was inferred by the observation, beginning in the 1970s, that patients who developed graft versus host disease (GVHD), particularly chronic GVHD, were relatively protected from disease relapse and had improved survival compared to patients without chronic GVHD (8, 9).

GVHD is a common and sometimes fatal complication of HSCT wherein donor immune cell alloreactivity against host tissues causes inflammation, leading to tissue injury (10). In its acute form, typically observed in the first 100 days after HSCT, the most common manifestations include diarrhea, rash, and liver function abnormalities (11). The chronic form of GVHD, usually developing or persisting after 100 days post-HSCT, has many protean manifestations but most commonly affects the skin, eyes, mouth, liver, fascia, and lungs (12). The pathogenesis of GVHD is incompletely understood (12, 13), and, although beyond the scope of the current review, has been well-reviewed elsewhere (14–21). As a major cause of treatment related morbidity and mortality, adequate prevention of GVHD is a key component of HSCT, and numerous strategies have been developed for GVHD prophylaxis (22). Since acute GVHD is thought to be driven largely by donor T cell alloreactivity (23–25), one early and highly effective strategy for GVHD prevention is T cell depletion (TCD) of allografts (26–31). While efficacious for preventing GVHD, many studies have found that TCD is associated with higher relapse rates, consistent with the role of T cells in driving both GVHD and GVL (3, 32–37). Thus donor immune cell activity (predominantly T cell alloreactivity) driving both GVHD and GVL are closely linked phenomena.

Recognition of the relationship of GVHD with GVL prompted early trials of adoptive cellular therapy for relapse prevention. In 1989, Sullivan et al. hypothesized that post-HSCT infusion of donor buffy coat (lymphocytes) to induce GVHD would likewise offer protection from relapse (38). Patients were randomized to receive standard GVHD prophylaxis alone or in combination with buffy coat lymphocytes from the stem cell donor, with the hypothesis that patients receiving the buffy coat lymphocytes may have reduced relapse risk through induction of GVHD. The incidence and severity of acute GVHD was much higher in the patients receiving buffy coat, as was non-relapse mortality (driven largely by infections complicating GVHD); however, 5-year relapse rates did not differ between the two cohorts. Curiously, this study did not find an increased incidence of chronic GVHD among recipients of buffy coat lymphocytes, perhaps explaining the absence of protection from relapse since other studies have consistently found a greater effect of chronic rather than acute GVHD associated with reduced relapse risk (39–42).

The first successful demonstration of DLI for treatment of relapsed disease was reported in 1990 by Kolb et al. (5) In this study, three patients with relapsed chronic myelogenous leukemia (CML) after HSCT were treated with DLI, resulting in complete hematologic and molecular remissions for all patients. Two of the patients developed GVHD. This report marked a turning point in the ability to leverage adoptive cellular immunotherapy for treatment of post-HSCT relapsed hematologic malignancies. A subsequent case report of one patient treated with DLI for post-HSCT relapsed CML demonstrated achievement of molecular remission (e.g. elimination of detectable Philadelphia chromosome BCR-ABL by polymerase chain reaction [PCR]) and return of full donor chimerism in the bone marrow; however, this was only achieved after onset of GVHD, underscoring the relationship between these phenomena and successful DLI (43).

## 3 Section 2 – clinical applications of DLI

### 3.1 DLI in CML and other diseases

Prior to 2000 and the subsequent approval of imatinib for treatment of CML, this disease was a common indication for HSCT; thus, many of the early studies of successful DLI were established in CML (5, 44). After 2000, CML became a less common indication for HSCT while the frequency of HSCT for AML increased (45). Several studies have established that post-HSCT relapsed CML is highly sensitive to the GVL effects of DLI, with complete response rates between 60-80% (46–49). DLI has less efficacy in other hematologic diseases, with response rates of approximately 20-30% for other diseases including acute myeloid leukemia (AML), acute lymphocytic leukemia (ALL), multiple myeloma (MM), and non-Hodgkin lymphomas (NHL) (46, 50, 51). Furthermore, CML patients who achieve complete molecular remission after DLI have highly durable responses, sometimes lasting several years and thus resulting in effective cures (52, 53).

Why CML harbors such exquisite sensitivity to the GVL effect of DLI is unclear. Yet this sensitivity to anti-leukemic alloreactivity receives support from several other lines of evidence. First, CML patients are most sensitive to the increased risk of relapse associated with T cell depleted HSCT; whereas T cell depleted HSCT for other diseases does not appear to impact relapse rate to the same degree (3, 34–36, 54, 55). Second, the relationship of development of chronic GVHD with reduced relapse risk appears to be stronger for CML compared to other diseases including AML (56, 57). Curiously, DLI appears to be even more effective for post-HSCT relapsed CML than imatinib (58), The relative homogeneity of stable phase CML compared to other hematologic malignancies could underlie differences in biologic responsivity to GVL effects of donor T cells. Interestingly, more blast phase CML, a more advanced phase biologically more similar to acute leukemia, is relatively insensitive to the GVL effect of DLI, further underscoring the so far undefined contribution of disease biology to success or failure of GVL (46, 59, 60). Yet the basis for these observations require further study which may illuminate opportunities to optimize DLI for other diseases through better understanding of the molecular mechanisms responsible for response to DLI in CML.

### 3.2 Dosing and interval of DLI

The initial paper reporting success of DLI administered total nucleated cells at a dose of approximately 4x10^8^ cells per kilogram (kg) of body weight, and while this strategy was successful in achieving remission, some patients developed GVHD (5). Subsequent studies sought to determine the optimal dosing for preservation of GVL while minimizing GVHD. One such study published in 1995 evaluated 22 patients with relapsed CML after HSCT who received DLI at 8 dose levels between 1 x 10^5^ and 5 x 10^8^ CD3+ T cells/kg in 4 to 33 week intervals (61). Eight patients achieved remission at doses as low as 1 x 10^7^ CD3+ cells/kg, while 11 additional patients required escalating doses between 5 x 10^7^ CD3+ cells/kg to 5 x 10^8^ CD3+ cells/kg. This group concluded that doses of 1 x 10^7^ cells/kg were a reasonable starting point, with the option of escalating doses with subsequent infusions to achieve remission. A later case report demonstrated achievement of remission in a patient with extensive GVHD with DLI doses of 1 x 10^6^ and 5 x 10^6^ CD3+ cells/kg (62). A larger study of 40 patients with relapsed CML treated with one of three different doses of CD4+ (i.e. CD8+ depleted) DLI: 0.3, 1.0, or 1.5 x 10^8^ cells/kg (63). Patients who failed to achieve remission at the first dose level could receive escalating doses in an attempt to achieve response. All four patients at the highest dose level achieved response, compared to 10 of 14 patients at the lowest dose level; however, the higher dose level had double the incidence of GVHD compared to the lower dose. Subsequent studies have confirmed these findings and suggested that DLI doses of 1 x 10^7^ cells/kg are sufficient to induce response with an acceptable rate of GVHD, while higher doses do not meaningfully increase response rates but do drastically increase the risk of toxicity (64).In the current era, DLI is most commonly given at a starting dose of 1 x 10^7^ cells/kg with an interval of approximately four weeks between infusions to allow for development of GVHD, per EMBT guidelines (65). Up to three or four total infusions may be administered. For prophylactic or pre-emptive indications, lower starting doses, such as 1 x 10^5^ or 1 x 10^6^ cells/kg may be considered.

### 3.3 Timing of DLI: prophylactic vs. preemptive vs. therapeutic

While DLI was initially developed as a therapy for relapsed disease (46), multiple studies have demonstrated across different diseases that DLI is most successful in reinstating remission in patients with lower disease burden (51, 66, 67). To improve likelihood of response, differences in timing of DLI have been explored.

#### 3.3.1 - Prophylactic DLI

Since relapse remains the leading cause of mortality (68), strategies for reducing relapse are paramount for improving overall outcomes for patients undergoing HSCT. Thus, administering DLI prophylactically, particularly for patients at higher than average risk of relapse and thereby capitalizing upon the additional GVL effects of adoptive cellular therapy presents an attractive option. Patients for whom prophylactic DLI is administered are typically without any signs of impending or molecular relapse (e.g. full donor chimerism, no decreasing blood cell counts or return of disease-defining mutations) and are typically treated within the first six months following HSCT. A registry study comparing matched pairs of patients who either did or did not receive prophylactic DLI showed that there was no difference in relapse rate across the entire cohort, patients with high-risk AML (i.e. unfavorable cytogenetic risk category or transplant in second complete remission or beyond) had improved overall survival if they received prophylactic DLI (**Table 1**) (69). Another retrospective analysis demonstrated a similar improvement in relapse rate for recipients of prophylactic DLI (22%) compared to the control group (53%) (70). Unsurprisingly, the major potential adverse event after DLI is development of GVHD (71, 74), which has been reported as being higher in prophylactic DLI compared to preemptive or therapeutic DLI (71). Several studies have also leveraged G-CSF priming of prophylactic DLI products, which may enhance GVL activity while mitigating GVHD through induction of immunomodulatory effects by G-CSF (72, 73, 75).

**Table 1:** Prophylactic DLI.

Leukemia Type(s)	Sample Size	Intervention	Comparison Groups	Response Rate	GVHD Rate	Reference
•AML •ALL	89 matched pairs	Prophylactic DLI from MRD or MUD	DLI vs no DLI	•No difference in OS overall •Improved OS for DLI recipients with high-risk AML	Incidence of: •aGVHD: 16% •cGVHD: 28%	•Schmid et al. (69)
•AML	46 cases, 34 controls	Adjuvant DLI in escalating doses	DLI vs no DLI	7-year OS: •67% for DLI •31 for no DLI	Incidence of: GVHD •aGVHD: 12% •cGVHD: 24%	•Jedlickova et al. (70)
•MDS/AML •ALL •HL •NHL •CLL •CML •MM	172	DLI from haploidentical donor	Prophylactic vs pre-emptive vs therapeutic DLI	OS after 1^st^ DLI: •61% for prophylactic •20% for pre-emptive •21% for therapeutic	High incidence of cGVHD in prophylactic (53%)	•Santoro et al. (71)
•AML •ALL	123	G-CSF primed DLI	DLI vs no DLI in high-risk acute leukemia	3-year OS: •36% for DLI •11% for no DLI	Incidence of: •aGVHD: 14% •cGVHD: 12%	•Wang et al. (72)
•MDS/AML •ALL •MM •MPN •NHL	44	G-CSF primed DLI	Steady state vs G-CSF primed DLI	Higher conversion to full chimerism and lower relapse for G-CSF primed DLI	Higher rates of cGVHD with G-CSF primed DLI	•Schneidawind et al. (73)

AML, acute myeloid leukemia; ALL, acute lymphoblastic leukemia; MDS, myelodysplastic syndrome; MPN, myeloproliferative neoplasm, MM, multiple myeloma; HL, Hodgkin lymphoma; NHL, non-Hodgkin lymphoma; CLL, chronic lymphocytic leukemia; CML, chronic myeloid leukemia; cGVHD, chronic graft versus host disease; G-CSF, granulocyte colony stimulating factor; MRD, matched related donor; MUD, matched unrelated donor; OS, overall survival.

#### 3.3.2 - Pre-emptive DLI

Hematologic or morphologic relapse of leukemia, defined as reappearance of greater than or equal to 5% bone marrow or peripheral blood blasts, can often be preceded by molecular relapse (i.e. recurrence of cytogenetic or molecular abnormalities associated with underlying disease) or other indications of impending relapse, including decreasing donor chimerism (76). Furthermore, presence of both mixed chimerism and measurable residual disease (MRD) after HSCT are both well-established predictors of later relapse, particularly for recipients of reduced intensity conditioning (RIC) HSCT (77–81). Therefore, administration of DLI at the first sign of impending or molecular relapse, termed preemptive DLI, has been evaluated as a therapy for leveraging GVL and re-establishing remission. Overall, preemptive DLI has a higher success rate compared to DLI for overt disease relapse, particularly for AML, with some studies reporting as high as 70% and five-year overall survival approaching 70% for AML (**Table 2**) (82–85). Advances in quantification of MRD before and after HSCT provide an opportunity to leverage GVL effects of preemptive DLI in this subset of patients at higher risk of relapse (91, 92). One early example of this was a study that utilized measurement of the Wilms tumor 1 gene (*WT1*), a commonly expressed AML-associated antigen, as a readout of MRD positivity post-HSCT (86). Patients who were MRD-positive for *WT1* received either preemptive DLI or low dose IL-2, with those receiving preemptive DLI having improved overall survival and lower incidence of relapse compared to the IL-2 group (86). A more recent study found a similar benefit of preemptive DLI on longer term clinical outcomes but also noted positive outcomes among patients receiving low dose IL-2 rather than DLI, suggesting this as a viable alternative for patients in whom DLI may be contraindicated (e.g. presence of GVHD) or infeasible (e.g. donor availability) (93). Aside from MRD positivity, the main indication for preemptive DLI is low or decreasing donor chimerism post-HSCT, which is a well-established predictive marker of later relapse, particularly for recipients of RIC regimens (77). Multiple studies have established that preemptive DLI is an effective strategy for improving donor chimerism post-HSCT, and that attaining full donor chimerism is linked to improved longer term overall survival and reduced relapse (87–90, 94).

**Table 2:** Preemptive DLI.

Leukemia Type(s)	Sample Size	Intervention	Comparison Groups	Response Rate	GVHD Rate	Reference
•MDS/AML	113	DLI after T-cell depleted HSCT	Pre-emptive vs therapeutic DLI	Pre-emptive DLI: •5-year OS 80% •5-year EFS 65% Therapeutic DLI: •5-year OS 40% •5-year relapse rate 69%	Pre-emptive DLI: •5-year cGVHD 31% Therapeutic DLI: •5-year cGVHD 45%	•Krishnamurthy et al. (82)
•AML •ALL	318	DLI for mixed chimerism or MRD	Descriptive	5-year outcomes: •CRI: 29% •NRM: 13% •LFS: 58% •OS: 64%	5-year outcomes: •cGVHD: 31%	•Schmid et al. (83)
•AML •ALL •CML	70	DLI for MRD in anticipation of relapse	Pre-emptive vs therapeutic DLI vs no DLI	1-year OS: •Pre-emptive: 94% •Therapeutic: 27% •No DLI: 65%	Incidence of aGVHD: •Pre-emptive: 63% •Therapeutic: 27%	•Tan et al. (84)
•MM	23	DLI for MM	Pre-emptive vs therapeutic DLI	Higher response rate for pre-emptive DLI	Incidence of Grade II-IV aGVHD: 22%	•Beitinjaneh et al. (85)
•MDS/AML •ALL	814	Risk-stratification DLI based on MRD	•709 MRD- (no tx) •49 IL-2 for MRD+ •56 IL-2 + DLI for MRD+	3-year CRI: •MRD-: 18% •MRD+, IL-2: 64% •MRD+, IL-2+DLI: 28%	Incidence of aGVHD: •MRD+, IL-2: 10% •MRD+, IL-2+DLI: 31% Incidence of cGVHD: •MRD+, IL-2: 37% •MRD+, IL-2+DLI: 43%	•Yan et al. (86)
•MDS/AML •MPN •CML •CLL •NHL •HL •MM •T-PLL	32-119	DLI for mixed chimerism	Descriptive	Pre-emptive DLI results in full donor chimerism and improved survival compared to historical controls	Incidence of GVHD: •Ref 87: 42% acute, 59% chronic •Ref 88: 67% •Ref 89: 73% •Ref 90: 35% overall, 17% acute, 22% chronic	•Solomon et al. (87) •Stadler et al. (88) •Caldemeyer et al. (89) •Feliu et al. (90)

MRD, measurable residual disease; OS, overall survival; EFS, event-free survival; CRI, cumulative incidence of relapse, NRM, nonrelapse mortality; LFS, leukemia free survival.

#### 3.3.3 - Therapeutic DLI

Therapeutic DLI, given for morphologic/hematologic relapse, is the most studied and commonly employed strategy. However, in sharp contrast to preemptive DLI, the success rates of therapeutic DLI for most diseases is quite poor, with the exception of CML (46). A nationwide Japanese study found that while patients given preemptive DLI for cytogenetic/molecular relapse (mostly AML) had a response rate of 57%, patients treated with therapeutic DLI after hematologic/morphologic relapse only had a 20% response rate, likely related to higher tumor burden and/or higher risk cytogenetic/mutational profiles, which is in line with response rates reported by other groups for DLI given in the setting of hematologic relapse (**Table 3**) (95, 98). Further, development of GVHD after DLI is linked to response, but in at least one study did not impact overall survival (63, 99). Some small studies, particularly in the CML setting have shown efficacy of a lower CD3+ starting dose for initial DLI infusion, which in some patients is sufficient to induce remission, later followed by escalating DLI doses, in an attempt to mitigate development of GVHD (96, 97, 100–102). The majority of this review will focus on applications of therapeutic DLI for relapsed disease.

**Table 3:** Therapeutic DLI.

Leukemia Type(s)	Sample Size	Intervention	Comparison Groups	Response Rate	GVHD Rate	Reference
•MDS/AML •ALL •CML •MM •NHL	140	Therapeutic DLI	Descriptive	•Best outcomes for patients with CML (60% CR, 2-year EFS 90%) •CR rate for AML 15%	Incidence of: •aGVHD: 60% •cGVHD: 61%	•Collins et al. (46)
•MDS/AML •ALL •CML •NHL •ATLL	414	Therapeutic DLI	DLI for cytogenetic or molecular vs hematologic relapse	100-day response rate: •57% for molecular relapse 20% for hematologic relapse	Incidence of GVHD: •Grade I: 7% •Grade II: 10% •Grade III: 6% •Grade IV: 6%	•Miyamoto et al. (95)
•CML	48	Escalating dose DLI	Bulk dose vs escalating dose	Similar remission rates across groups	Lower GVHD with escalating dose	•Dazzi et al. (96)
•MM •HL •NHL	49	Therapeutic DLI	Descriptive	Response rate of 63% for MM and 70% for HL, not predicted by change in chimerism	24% incidence of Grade II-IV GVHD (more common after unrelated donor DLI)	•Peggs et al. (97)

CR, complete response; ATLL, adult T leukemia/lymphoma.

#### 3.3.4 - DLI versus second HSCT

For patients with overt relapse of disease after first HSCT, there is no established therapy with superior response rates, though options include additional therapy (chemotherapy, hypomethylating agents), DLI, second HSCT, or therapy followed by DLI or second HSCT. There is clinical equipoise surrounding the question of whether DLI or second HSCT is the superior strategy. A large EBMT registry study of 418 adults with relapsed AML assessed outcomes after DLI compared to second HSCT. No difference in overall survival was observed between the two treatment groups; rather, outcomes were better, irrespective of cellular therapy approach, for patients who relapsed >6 months after first HSCT compared to those who relapsed early (<6 months) after HSCT (103). Another smaller retrospective study analyzed outcomes for 89 patients with relapse or graft failure who received either DLI or second HSCT and found a trend toward improved overall survival for recipients of second HSCT. Only selected patients may be candidates for either strategy. In general, patients must have no or minimal GVHD present for consideration of DLI or second HSCT. Additionally, older and less fit patients may be recommended for DLI rather than HSCT if they are not deemed to be sufficiently fit to tolerate conditioning therapy ahead of a second HSCT. Thus, rigorous comparison of these two interventions is difficult, and the decision of which strategy to employ remains a highly individualized process, dependent upon the specific patient, disease, and toxicity characteristics in each case (104).

### 3.4 Manipulations to DLI

#### 3.4.1 - CD8 depletion

Various strategies have been employed to optimize GVL response of DLI and/or minimize toxicities of associated GVHD through manipulation of the DLI product (**Table 4**). One early strategy hypothesized that, since some evidence implicated CD8+ T cells in the pathogenesis of GVHD (131), perhaps infusion of CD4+ T cells alone would result in effective GVL without GVHD (63). Patients with relapsed CML, MM, or other diseases were treated with defined doses of CD8-depleted CD4+ T cells with clinical responses in ~80% of patients and GVHD developing in about one-third of patients [compared to a ~75% GVHD incidence with conventional DLI (46)]. With this encouraging result, several additional studies evaluated CD8-depletion as a strategy for preserving GVL while reducing GVHD for not only DLI for disease relapse (30, 46, 105), but also as a prophylactic strategy for patients receiving RIC HSCT (132) or those with mixed chimerism (MC) or persistent disease immediately following HSCT (106). However, this latter study did still find a high incidence of grade II-IV acute GVHD (5 of 22 patients, 23%), suggesting that CD8 T cells are not the sole drivers of GVHD. Indeed, subsequent work has drawn a clear link between CD4+ T cell activity and GVHD development. These studies all demonstrated encouraging data for GVHD incidence with preserved GVL activity, including sustained remission for the majority of patients (133). Studies in mice have demonstrated that naive CD4+ T cells alone are capable of inducing GVHD, while mice receiving memory CD4+ T cells are protected from GVHD (134–137). Several murine studies have established the ability of CD4+ T cells to promote GVHD, for example through major histocompatibiltiy complex (MHC) II presentation of intestinal microbiome antigens or other non-hematopoietic recipient antigens to promote GVHD (138–142).

**Table 4:** Strategies for GvL manipulation or augmentation.

T4Effector Cell Type/Manipulation	Disease/stage	Treatment Setting	Response Rate	GvHD Rate	Reference(s)
Unmanipulated DLI	•MDS/AML •ALL •CML •MM •NHL	•Therapeutic DLI	•Best outcomes for patients with CML (60% CR, 2-year EFS 90%) CR rate for AML 15%	Incidence of: •aGVHD: 60% cGVHD: 61%	•Collins et al. (46)
CD8 Depletion	•CML in remission (30) •Relapsed CML, MM, CLL, NHL or MDS (63, 105) •Persistent disease or mixed chimerism (FL, CLL, HL, MCL, MM, MF, AML (106)	•Prophylactic (30) •Therapeutic (63, 105) •Pre-emptive (106)	50-89%	0-30%	•Soiffer et al. (30) •Alyea et al. (63, 105) •Orti et al. (106)
Naïve T cell Depletion	•AML, ALL, other malignancy, BM failure or immunodeficiency (107) •AML, NHL, MM, HL, CLL, MDS/MPN (108–110)	•Prophylactic DLI (108–111) •Therapeutic DLI (112)	50-80%	2-19%	•Maschan et al. (108) •Maung et al. (109) •Dunaikina et al. (110) •Castagna et al. (111) •Muffly et al. (112)
Treg Depletion	•AML, ALL, NHL, HL (113) •AML, NHL, MM, HL, NHL, CLL, MDS (114)	•Therapeutic DLI	40-50%	33-40%	•Nikiforow et al. (113) •Maury et al. (114)
Alloanergization	•MDS/AML	•Prophylactic DLI	–	29%	•Davies et al. (115)
Donor Cell Preactivation – IL-2	•AML, ALL, CML	•Therapeutic DLI	59%	30%	•Slavin et al. (116)
Donor Cell Preactivation – Cytokine-Induced Killer Cells	•MDS/AML, ALL, CLL, MM, HL, NHL (117, 118) •Myeloid neoplasm (119)	•Therapeutic (120, 121) •Pre-emptive (121, 122)	30-53%	17-25%	•Laport et al. (117) •Narayan et al. (119) •Merker et al. (118)
Leukemia Antigen Vaccination	•HL, CLL, MF, NHL, AML, MDS, MM (123) •AML (124)	•Therapeutic DLI	29-71%	12%	•Ho et al. (123) •Rosenblatt et al. (124)
DLI+Azacitidine	•MDS/AML	•Therapeutic (125–128) •Prophylactic (128) •Pre-emptive (129, 130)	33-70%	0-45%	•Tessoulin et al. (125) •Schroeder et al. (126) •Ghobadi et al. (127) •Guillaume et al. (129) •Rautenberg et al. (130) •Liberatore et al. (128)
Cytokine-Induced Memory-Like NK cells	•Myeloid neoplasm	•Therapeutic DLI	56%	0%	•Romee et al.

AML, acute myeloid leukemia; ALL, acute lymphoblastic leukemia; MDS, myelodysplastic syndrome; MPN, myeloproliferative neoplasm; MM, multiple myeloma; HL, Hodgkin lymphoma; NHL, non-Hodgkin lymphoma; CLL, chronic lymphocytic leukemia; CML, chronic myeloid leukemia; FL, follicular lymphoma; MF, myelofibrosis; MPAL, mixed phenotype acute leukemia; BPDCN, blastic plasmacytoid dendritic cell neoplasm cGVHD, chronic graft versus host disease.

#### 3.4.2 - Naive T cell depletion

Several groups have posited that GVHD pathogenesis is driven largely by alloreactive naive T cells and hypothesized that selective depletion of CD45RA+ naive T cells may reduce GVHD while retaining GVL, a strategy that has been applied to HSCT with encouraging results (107, 143–145). To this end, several groups have piloted methods for efficiently depleting CD45RA+ cells from HSCT followed by prophylactic DLI with T cells depleted of the naive compartment for reduction in GVHD with the aim to preserve GVL (108–111). These relatively small studies have indicated safety and efficacy of this approach. One study of 15 patients has applied this approach for therapeutic DLI for treatment of relapsed disease with donor-derived CD8+ memory T cells, showing safety and low incidence of GVHD (112).

#### 3.4.3 - Treg depletion

There is considerable evidence in both mice and humans that prevalence of CD4+CD25+ regulatory T cells (Tregs) is associated with protection from GVHD, and these cells may play an important role in mitigating GVHD pathogenesis through control of effector T cell alloreactivity (146–151). It has been hypothesized that Tregs may also dampen GVL through a similar mechanism (152). This idea received some support from the finding that patients who achieved durable remission after DLI for treatment of relapsed disease had lower proportion of Tregs in the DLI product compared to patients who did not respond (153). Two independent groups subsequently sought to explore this concept further in clinical trials testing the safety and efficacy of DLI depleted of Tregs (113, 114) Maury et al. studied 17 patients with relapsed disease who had failed to respond conventional DLI. Two patients experienced disease response to a single infusion of Treg-depleted DLI, while an additional four patients responded after a second infusion of DLI. All responders experienced GVHD (114). Longer term follow up demonstrated that three of the six responders remained in remission at 5 years following Treg-depleted DLI, while three patients who initially had a response ultimately relapsed with the original malignancy and succumbed to disease within three years of treatment (154). The Dana-Farber group conducted a phase I trial of 21 patients treated with CD25+ Treg-depleted DLI at two different dose levels, of whom four were in complete remission at the time of DLI after treatment with cytoreductive therapy for relapse (113). At the lower dose (1x10^7^ CD3+ cells/kg), one of six patients responded, while at the higher dose (3x10^7^ CD3+ cells/kg), 60% experienced complete or partial response, with a one year overall survival rate in that cohort of 53%. Seven patients developed GVHD, one of whom died as a result of this complication. Comparison to a contemporaneous cohort of conventional DLI therapy suggested improved response rates with Treg depletion (113).

#### 3.4.4 - Alloanergization

Alloanergization is an approach to prevent GVHD by tolerizing alloreactive donor T cells to the recipient through chronic antigen stimulation with concurrent co-stimulatory blockade, thus leading to T cell anergy and hyporesponsiveness to alloantigens (155). Initially developed as a strategy to expand the pool of potential donors beyond full HLA-match, alloanergization has been shown to be an effective method of administering CD34+-selected haploidentical HSCT, followed by DLI, with *in vitro* studies suggesting intact GVL (115, 156, 157).

#### 3.4.5 - Activation of donor cells

Early experiments in mouse models of leukemia and HSCT suggested that activation of alloreactive donor T cells could prevent or treat relapse through enhanced GVL (158). These observations prompted the hypothesis that promoting T cell activation with cytokines such as IL-2 *ex vivo* or *in vivo* prior to DLI could improve outcomes (159, 160). A case report of a patient with relapsed CML after HSCT lended support for this approach, when the patient achieved remission after three sequential DLI treatments, along with subcutaneous injections of recombinant IL-2 (161). A subsequent larger phase I study in patients with relapsed acute or chronic leukemia (ALL, MDS/AML, or CML) further supported this notion, wherein more than half of the patients demonstrated response to combination therapy (116). Interestingly, in this study, six of the 10 responding patients developed GVHD, while in four patients GVL was independent of GVHD. Only one of the seven nonresponder patients developed GVHD, underscoring the close relationship between these immunologic phenomena. Another strategy for is using *ex vivo* cytokine treatment to activate donor T cells, thereby priming their killing efficacy (cytokine-induced killer [CIK] cells) (117). Two groups have recently tested the efficacy of CIK cells to carry out GVL activity. The first group administered a single infusion of CIK to patients who did not achieve full donor chimerism after HSCT, since these patients are at higher risk of relapse (119). CIK infusion did not appear to substantially impact donor chimerism at day +90 after HSCT (~55-70 days after CIK), and outcomes including survival and relapse rates were similar to historical cohorts. The second group tested conventional DLI compared to CIK infusion in patients with molecular or hematologic relapse after HSCT (118). In this study, all patients with hematologic relapse progressed after DLI or CIK; however, among patients with molecular relapse only, complete remissions were induced in 53% of patients treated with CIK versus 29% with conventional DLI. This promising result suggests that CIK improves the GVL activity of donor cells over conventional DLI and may be an effective therapy for molecular relapse of disease.

#### 3.4.6 – Leukemia antigen vaccination

Other studies have questioned whether the GVL effect of DLI can be promoted by activation of alloreactive T cells in an antigen specific way through individualized vaccination strategies. While vaccination has been employed for solid tumors including melanoma and renal cell carcinoma with some degree of success (162–164), hematologic malignancies generally are characterized by lower tumor neoantigen burden, thus decreasing the likelihood of antigen-specific T cell responses (165). Nevertheless, other potential antigens including leukemia-associated antigens or minor histocompatibility antigens (mhAgs) in the post-HSCT setting may be targets (166, 167). To this end, a phase I clinical trial tested whether infusion of donor-derived dendritic cells (DCs) cultured in the presence of GM-CSF and IL-4 could enhance the GVL effect of DLI (123). The study found that this was a safe and feasible approach for modifying DLI, and that four of the patients achieved a durable remission lasting greater than 5 years. A similar vaccination strategy was tested at the time of HSCT (rather than for post-HSCT relapsed disease) wherein personalized vaccines were created for patients with AML undergoing HSCT (124). In this study, donor-derived DCs generated from PBMCs were cultured with GM-CSF and IL-4 along with AML cells derived from the patient’s original diagnosis. Patients were vaccinated serially after HSCT, which was generally well tolerated and resulted in 70% overall survival at nearly 5 years post-HSCT. Correlative analysis demonstrated rise and persistence of circulating T cells reactive to whole AML cells and leukemia-associated antigens. Despite these encouraging results, a subsequent phase II randomized study of inoculation with similarly generated leukemia-reactive DC vaccines compared to placebo showed no difference in overall survival, progression-free survival, or relapse (168). Altogether, antigen-specific activation of alloreactive donor T cells is an attractive possibility for enhancing GVL, though the optimal strategy requires further study.

#### 3.4.7 Interferon to stimulate GVL

Treatment with interferon alpha (IFNɑ) has been investigated, either alone or in combination with DLI, as a potential method for promoting GVL activity. The basis for this hypothesis lies in the potential ability of IFNɑ to stimulate expression of cell surface molecules on leukemia cells, thereby sensitizing leukemia cells to alloimmune activity, while IFNɣ contributes to regulation of T cell differentiation and function (120, 122). IFNɑ may also aid in preventing GVHD by inhibiting CD4 proliferation (121). One early study treated 18 patients with IFNɑ prior to DLI and found that four of 14 assessable patients successfully engrafted with donor cells (169). A subsequent small comparative study assessed response to DLI alone in 3 patients versus DLI with IFNɑ in 10 patients, where 9 of 10 patients treated with DLI and IFNɑ achieved complete molecular remission whereas all of the patients treated with DLI alone experienced disease progression (170). DLI with interferon appears to be effective in CML (66), though small studies in MDS/AML have shown promise, though this strategy has not been evaluated in larger randomized clinical trials (171, 172). Interferon gamma (IFNɣ) has also been studied as a strategy to promote GVL, since T cell secretion of IFNɣ can have anti-tumor effects (173). Murine studies of IFNɣ wild-type or knockout mice have shown that IFNɣ deletion leads to augmentation of GVHD in an allogeneic transplant model, with GVL effects inversely correlated with GVHD (174). A subsequent study demonstrated the ability of IFNɣ to promote GVL in the context of interferon gamma receptor knockout leukemia cells, indicating that the anti-leukemic properties of IFNɣ did not require direct interaction with the leukemia cells themselves. Though a direct anti-leukemic function of IFN may not be required for effective GVL, IFNɣ has been shown to upregulate expression of MHC class II genes in AML and blast crisis CML cells, whereas chronic phase CML cells were sensitive to GVL effects independent of IFNɣ (175). A recent phase I clinical trial in humans have leveraged this effect by combining IFNɣ treatment with DLI, with promising safety data as well as evidence of increased MHC class II expression and improvement in T cell chimerism in four patients with relapsed MDS/AML (176, 177). Subsequent larger studies are needed to determine the clinical efficacy of this approach.

### 3.5 Clinical trials of DLI and combination therapy

Since patients have the greatest likelihood of responding to DLI when disease burden is lowest (51), several studies have attempted combination or sequential cytoreductive or targeted therapies with DLI to improve response rates. One prospective study evaluated 65 patients with relapsed myeloid malignancy who were treated with cytarabine-based chemotherapy followed by DLI (178). Approximately half of the 57 evaluable patients experienced response to therapy; however, treatment related mortality was high (23%), and 2-year overall survival was 19%. Among patients who experienced complete response after combination therapy, 2 year overall survival was 41%, indicating that achieving a response with chemoimmunotherapy is capable of leading to durable remissions.

More recently, several groups have evaluated the efficacy of hypomethylating agents, often azacitidine (Aza), in combination with DLI for treatment of relapse after HSCT (**Table 5**). A 2014 study examined outcomes of 31 patients with relapsed myeloid malignancy after HSCT treated with Aza salvage therapy, of whom 11 received at least one DLI in addition (125). Response rate was 35% (14% complete response, 21% stable disease), but addition of DLI did not appear to influence likelihood of response. This study supported the idea of Aza as effective salvage therapy for relapsed myeloid malignancy, leading for subsequent larger studies, though the additive benefit of DLI remained unclear. A 2015 retrospective study from the German Cooperative Transplant Study Group evaluated outcomes of 154 patients with post-HSCT relapsed myeloid disease (AML, myelodysplastic syndrome [MDS], or myeloproliferative neoplasm [MPN]) of whom 105 received Aza followed by DLI while 49 received Aza alone. Thirty-three percent of patients had a response (27% with complete response, 6% with partial response), and patients with molecular-only relapse or MDS were more likely to be responders (126). Based on data from mouse studies demonstrating that azacitidine administration preserved GVL activity while preventing GVHD through immunomodulation of T cells (179, 180), a small phase I dose escalation study tested the safety and efficacy of DLI followed by Aza in the first 10 days, based on studies in mice that this strategy increased Treg numbers to provide protection from GVHD without loss of GVL (127). Six of the eight patients responded to treatment with no development of grade III-IV acute GVHD. A more recent study retrospectively analyzed 77 patients with high risk for relapse (54 with AML, 23 with MDS; high risk defined by genetic mutations or disease status at HSCT [>2nd complete remission or refractory disease]) who were treated with prophylactic low-dose Aza (up to 12 cycles if tolerated) followed by up to 3 doses of DLI after HSCT (129). Seventy-nine percent of patients ultimately received combination therapy, and overall survival at 2 years was 70%. Historial outcomes for patients with similarly high-risk disease demonstrate a ~20-53% 2-year overall survival rate (181–183). Another 2021 study measured expression of the MDS/AML-associated antigen WT1 as an indicator of MRD and therefore molecular relapse in 35 patients and then treated these patients with Aza and DLI (130). More than half of patients responded (37% MRD negative complete response, 20% MRD positive complete response, and overall survival at 2 years was 35%, supporting Aza and DLI combination therapy for treatment of early relapse. More recently, Aza and DLI, particularly for pre-emptive treatment of relapse, have received further experimental support. A multi-center retrospective analysis of 71 patients with myeloid neoplasms were treated with Aza alone or Aza and DLI (128). The overall response rate was 49%, with 38% of patients achieving complete response. This study reported that addition of DLI to Aza enhanced overall survival and event free survival; however, the main reason that patients may not proceed to DLI after Aza was disease progression (17 of 31 patients receiving Aza alone), possibly confounding the interpretation of the additive benefit of DLI to Aza. Nevertheless, combination of Aza and DLI appears to be a feasible strategy for leveraging enhanced GVL for pre-emptive treatment of MDS/AML relapse and may be a reasonable salvage treatment option for patients with hematologic relapse.

**Table 5:** DLI with HMA.

DLI Indication	Leukemia Type(s)	Sample Size	Intervention	Comparison Groups	Response Rate	GVHD Rate	Reference
Relapse (therapeutic)	•MDS/AML	31	Aza +/- DLI	Descriptive	•CR rate: 13% •Overall response: 35%	One reported case of aGVHD after DLI (out of 12 patients)	•Tessoulin et al. (125)
Relapse (therapeutic)	•MDS/AML	154	Aza +/- DLI	Molecular vs hematologic relapse; MDS vs AML	Improved outcomes for molecular only relapse or MDS vs hematologic relapse or AML	Incidence of: •aGVHD: 31% in patients receiving DLI •cGVHD: 31%	•Schroeder et al. (126)
Relapse (therapeutic)	•AML	8	Dose finding study for Aza + DLI	Descriptive	6/8 patients with response	5/8 patients with aGVHD (Two Grade 1, three Grade 2)	•Ghobadi et al. (127)
Prophylactic or Pre-emptive	•MDS/AML	77	Low dose Aza +/- dose escalated DLI	Descriptive	2-year outcomes: •CIR: 22% •OS: 71% •PFS: 68%	2-year incidence of: •aGVHD: 27% •cGVHD: 45%	•Guillaume et al. (129)
Pre-emptive	•MDS/AML	35	HMA + DLI for MRD detected by WT1	HMA + DLI vs no treatment	•37% major response •20% minor response •35% 2-year OS	Incidence of: •aGVHD: 18% •cGVHD: 41%	•Rautenberg et al. (130)
Relapse (therapeutic)	•MDS/AML	71	Aza +/- DLI for relapse after alternative donor HSCT	Aza alone vs Aza +/- DLI; molecular relapse vs hematologic relapse	Improved response rates for Aza + DLI vs Aza alone and for molecular relapse vs hematologic relapse	•1-year late aGVHD rate: 27% •1-year cGVHD rate: 18%	•Liberatoire et al. (128)

Aza, Azaciditine; PFS, progression-free survival; WT1, Wilms’ tumor 1; HMA, hypomethylating agent.

There is considerable interest in utilizing other strategies to enhance the immunologic effects of GVL for hematologic diseases, particularly MDS/AML, for example with immune checkpoint blockade (ICB), yet success of these approaches has been modest thus far (184). A recent phase I study demonstrated marked response of relapsed myeloid malignancies to ICB with the CTLA-4 inhibitor ipilimumab (185). Response was associated with enhanced cytotoxic CD8 T cell infiltration into leukemic tissues, with decreased Treg activation, and expansion of effector T cells in the blood of these patients, suggesting enhanced GVL activity. Subsequent molecular analysis of responding patient samples demonstrated increased expression of T cell activation and proinflammatory cytokines (186). This raises the interesting hypothesis that ipilimumab functions to restore T cell GVL activity and perhaps combination of ipilimumab with DLI could further amplify the GVL effect of DLI. A phase I trial is currently underway to test this hypothesis (NCT03912064).

### 3.6 Other cell types with putative GVL activity

Several nonclassical T cell subsets have also been implicated in GVL activity of DLI. Mouse studies have demonstrated that HSCT performed with donors lacking ɣδ T cells have impaired GVL activity and worsened GVHD (187). Studies of human primary cells *in vitro* have demonstrated a direct cytotoxic effect of ɣδ T cells on AML cells (188). One case report has implicated persistence of a donor-derived ɣδ T cell clone with prolonged survival after DLI for T-ALL (189). Invariant NKT cells (iNKT) are a rare CD1d-restricted T cell subset with phenotypic and functional characteristics shared between NK and T cells (190). *In vitro* studies using both leukemia cell lines and primary human AML blasts has demonstrated direct killing of leukemia cells in a CD1d-dependent manner by iNKT cells, an intriguing finding that warrants further mechanistic investigation (191).

Though the GVL effect of DLI is thought to be predominantly driven by alloreactive donor T cells, other immune cell types may play important roles in recognizing and eradicating malignant cells. NK cells are a population of innate lymphoid cells that normally function to eliminate viruses but may also have anticancer functions (192). NK cells treated with a cocktail of cytokines have been shown to acquire a memory-like state, endowing them with the ability to proliferative and carry out cytotoxic functions (193). These cytokine-induced memory (CIML) NK cells have been shown to have reactivity and cytotoxicity against myeloid leukemia cells in both mice and humans (194). An ongoing phase I trial aims to test infusion of CIML NK cells for treatment of relapsed myeloid disease after HSCT (NCT04024761). Preliminary correlative analysis of patient samples from this trial indicate that CIML NK cells rapidly expand and persist for months after infusion (195). Further study is needed to confirm the safety and efficacy of CIML NK adoptive cellular immunotherapy.

B cells have also been linked to GVL responses after DLI. One study identified the presence of antibodies that recognized CML-associated epitopes in the sera of patients who responded to DLI, and antibody titers were temporally associated with response (196, 197). A subsequent study of patients with CLL who received DLI for relapsed disease post-HSCT identified high titers of antibodies directed against CLL-associated antigens (198). Antibodies directed to Y chromosome antigens (H-Y antigens) in the setting of sex-mismatched HSCT have previously been implicated in both GVHD and GVL (199, 200). One study identified a coordinated B- and T-cell response directed toward the DBY antigen on the Y chromosome in a male HSCT recipient from a female donor (201). While the exact role of B cells in promoting the GVL effect remains unknown, these findings support the potential for B cell mediated cellular therapies or antibody therapy to enhance GVL.

## 4 Section 4 - mechanisms of response and relapse

### 4.1 - Immune escape leading to relapse

Relapse remains the leading cause of morbidity and mortality for patients post-HSCT, particularly for AML. In recent years, mechanisms of relapse, and in particular, mechanisms of immune escape from donor immune cell-mediated GvL are increasingly being recognized. A 2009 study of relapse after haploidentical HSCT identified loss of the mismatched HLA locus in leukemic cells in 5 patients after relapse because of uniparental disomy of chromosome 6p (202). This group demonstrated that donor-derived T cells were unable to recognize the relapsed mutant leukemia cells lacking the mismatched HLA haplotype, though donor T cells maintained the ability to recognize and mediate cytotoxicity toward leukemia cells from the original diagnosis that retained the mismatched HLA. This and other studies established HLA loss and uniparental disomy as an important mechanism of immune escape leading to leukemia relapse and resistance to maintenance of GvL (202, 203). In the HLA-matched setting, recurrent HLA loss and downregulation of MHC class II genes have also been identified as a mechanism of leukemic escape and relapse, likely due to loss of leukemia-associated antigens and/or mHAgs (204, 205). Subsequent studies have identified immunologic signatures associated with leukemia escape and relapse, including deregulation of co-stimulatory ligands (e.g. CD80, PD-L1), loss of response to GVL (IFNɣ) and tumor necrosis factor-ɑ (TNF-ɑ), and IL-2/STAT5 signaling (202, 206, 207). Strategies for employing DLI or other immune mediated GvL strategies will need to account for HLA-loss and the likelihood of effective GvL for each treatment strategy in light of these immune escape mechanisms sometimes employed by leukemic cells.

### 4.2 - GVL mechanisms of DLI

**Figure 1:**
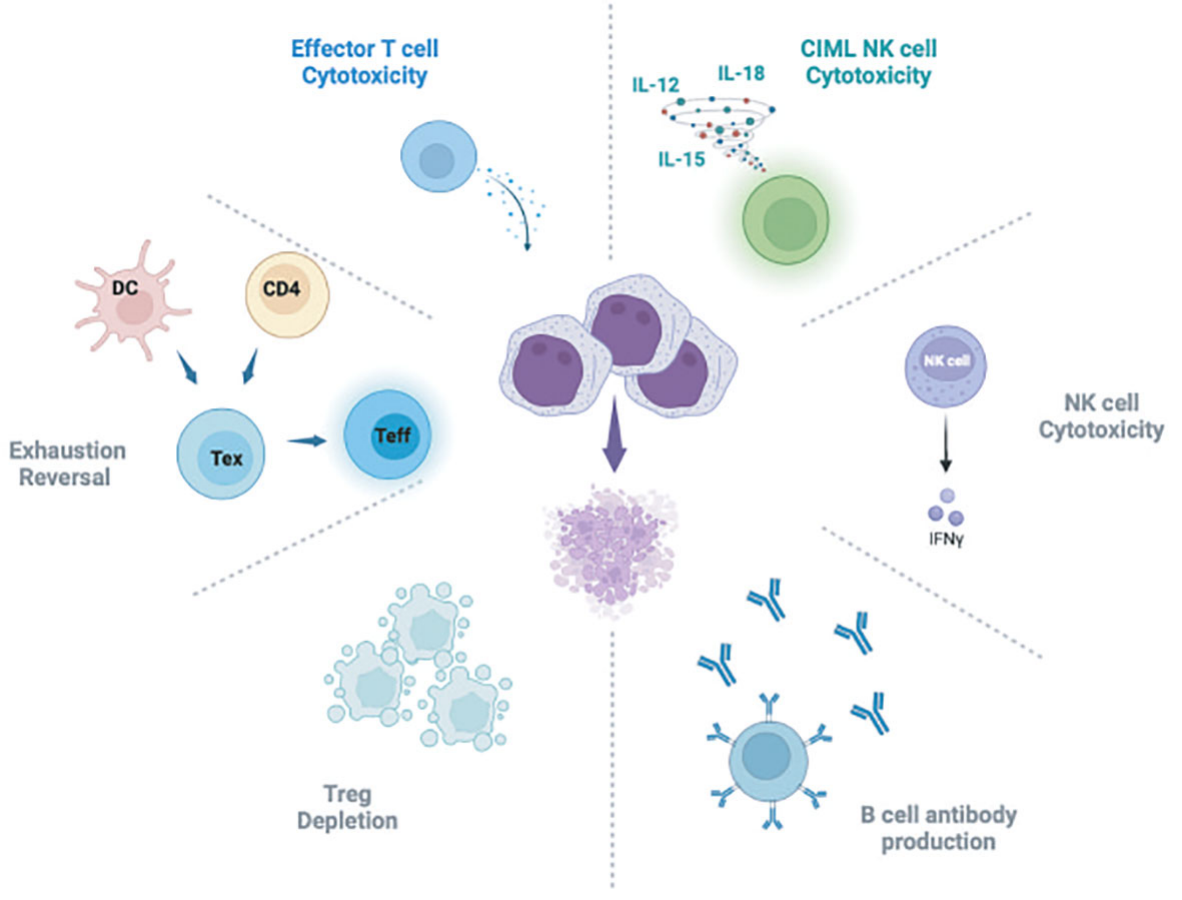
Schematic of hypothesized mechanisms of GVL activity attributed to DLI. DC, Dendritic cell; Tex, T exhausted cells; Teff, T effector cells; CIML NK, Cytokine induced memory-like NK cells. Figure generated using BioRender software.

Relapse of hematologic malignancy after HSCT may be at least in part attributable to development of T cell dysfunction due to exhaustion and therefore loss of the protective GVL effect endowed by HSCT. T cell exhaustion is well documented in viral infections and various cancer settings and is hypothesized to arise at least in part due to chronic antigen stimulation, though the pathways leading to exhaustion, particularly in the cancer setting, remain incompletely defined (208). ICB is thought to be effective as cancer therapy due to the ability to reverse T cell exhaustion, perhaps in specific “progenitor-exhausted” subsets, defined by expression of TCF1 (209, 210). More recently, single cell analysis of patients with post-HSCT relapsed CML treated with DLI demonstrate expansion of progenitor exhausted T cells among responding patients after DLI (211). Correlative studies of bone marrow samples from patients who received DLI for relapsed CML have identified enhanced infiltration of CD8 T cells and reversal of exhaustion in patients with effective GVL after DLI, in a mechanism similar to that seen in other tumor settings with ICB (212). Prior studies of patients with relapsed myeloid malignancy treated on a clinical trial with ipilimumab demonstrated similar CD8 T cell infiltration and exhaustion reversal in disease biopsies (185, 186). It has been hypothesized that CTLA-4 inhibition with ipilimumab may act on CD4 T cells, dendritic cells, or even perhaps AML leukemia cells themselves to contribute to reversal of exhaustion in CD8 effector T cells (185, 186, 213). T cell exhaustion reversal has also been implicated in AML responders to DLI (214). These data support a central role for reversal of exhaustion in the leukemic microenvironment as necessary for effective GVL after DLI. Further work is needed to clarify the mechanisms of T cell exhaustion reversal associated with effective GVL to better leverage this for improved future therapies (**Figure 1**).

As discussed above, many studies implicate immune responses to mHAgs in the GVL immune response (166, 215, 216). Several specific alloantigens have been identified that are capable of eliciting either CD4 or CD8 GVL immune responses (217–220). One correlative study of effector T cells derived from CML patients experiencing effective GVL after DLI demonstrated enhanced interferon gamma production from CD8 effector T cells, including cytotoxic T cells specific to patient-derived mhAgs (221). Though the precise antigens and mechanisms of GVL activity remain incompletely defined, some potential activation ligands have been identified. For example, stimulation of toll-like receptors (TLR) has been shown to be associated with GVL response after DLI (222, 223). In another murine model of HSCT, production of interleukin 12 (IL-12) by plasmacytoid dendritic cells (pDCs) was found to be associated with more effective GVL compared to mice receiving unmanipulated marrow enriched for conventional myeloid dendritic cells (224). The precise cellular and molecular mechanisms underlying effective GVL are likely complex and multifactorial. While major advances in molecular understanding have been made with the advent of more sensitive analytic modalities, additional research is needed to better define the key players and strategies for optimizing the GVL activity of DLI. 

## 5 Section 5: concluding remarks

The GVL effect associated with DLI for relapsed hematologic disease after HSCT is a clinically well-established phenomenon; yet, response rates for most diseases remain low. Future studies are critically necessary for determining optimal timing of DLI as well as strategies for enhancing GVL activity. While newer combination therapies with hypomethylating agents such as Aza or immune checkpoint inhibitors like ipilimumab have shown promise, further experimental evidence is needed to improve response rates and prevent or treat relapsed disease. Further, an improved molecular understanding of the precise cellular subsets and mechanisms underlying effective GVL as well as mechanisms of resistance to therapy will support development of rationally designed and more targeted therapies to sharpen the GVL effect of DLI while mitigating toxicities.

## Author contributions

KM: Conceptualization, Writing – original draft, Writing – review & editing. JA: Conceptualization, Writing – original draft, Writing – review & editing.
